# UV-Assisted 3D Printing of Polymer Composites from Thermally and Mechanically Recycled Carbon Fibers

**DOI:** 10.3390/polym13050726

**Published:** 2021-02-27

**Authors:** Andrea Mantelli, Alessia Romani, Raffaella Suriano, Marco Diani, Marcello Colledani, Essi Sarlin, Stefano Turri, Marinella Levi

**Affiliations:** 1 Department of Chemistry, Materials and Chemical Engineering “Giulio Natta”, Politecnico di Milano, Piazza Leonardo da Vinci 32, 20133 Milano, Italy; andrea.mantelli@polimi.it (A.M.); alessia.romani@polimi.it (A.R.); stefano.turri@polimi.it (S.T.); marinella.levi@polimi.it (M.L.); 2 Department of Mechanical Engineering, Politecnico di Milano, Via La Masa 1, 20156 Milano, Italy; marco.diani@polimi.it (M.D.); marcello.colledani@polimi.it (M.C.); 3 Unit of Materials Science and Environmental Engineering, Tampere University, Korkeakoulunkatu 6, 33720 Tampere, Finland; essi.sarlin@tuni.fi

**Keywords:** polymer-matrix composites (PMCs), crosslinking, resins, additive manufacturing, recycling, carbon fibers, rheology, mechanical testing

## Abstract

Despite the growing global interest in 3D printed carbon fiber reinforced polymers, most of the applications are still limited to high-performance sectors due to the low effectiveness–cost ratio of virgin carbon fibers. However, the use of recycled carbon fibers in 3D printing is almost unexplored, especially for thermoset-based composites. This paper aims to demonstrate the feasibility of recycled carbon fibers 3D printing via UV-assisted direct ink writing. Pyrolyzed recycled carbon fibers with a sizing treatment were firstly shredded to be used as a reinforcement of a thermally and photo-curable acrylic resin. UV-differential scanning calorimetry analyses were then performed to define the material crosslinking of the 3D printable ink. Because of the poor UV reactivity of the resin loaded with carbon fibers, a rheology modifier was added to guarantee shape retention after 3D printing. Thanks to a customized 3D printer based on a commercial apparatus, a batch of specimens was successfully 3D printed. According to the tensile tests and Scanning Electron Microscopy analysis, the material shows good mechanical properties and the absence of layer marks related to the 3D printing. These results will, therefore, pave the way for the use of 3D printed recycled carbon fiber reinforced polymers in new fields of application.

## 1. Introduction

Over the last decade, the use of 3D printing to manufacture thermoplastic composites has made remarkable progress in terms of processable materials, printability, and types of loaded fillers [1,2]. To date, a variety of polymer composites have been 3D printed to meet the requirements of a huge range of applications such as healthcare [3], aerospace and automotive sectors [4,5], textile and fashion-related fields [6], and packaging [7]. Among these, polymer composites loaded with carbon fibers (CFs) stand out for their good mechanical properties, and they have been recently 3D printed using a thermoplastic matrix by the technology of large format additive manufacturing [8]. When compared to other conventional technologies, the additive manufacturing of fiber-reinforced polymers offers the added advantage of providing desired complex shapes and geometries to obtain lightweight and robust objects without heavily increasing the production costs [9].

In addition to 3D printed thermoplastic polymers reinforced with short and continuous CFs [10,11], UV-assisted three-dimensional (UV-3D) printing technology has been recently used to build 3D scale models of complex structural components such as airfoils and propellers composed of CF reinforced thermosetting polymers [5]. Using the same approach, 3D freeform and freestanding nanocomposites were successfully fabricated to produce cellular nanocomposite materials with hexagonal or squared unit cells [12]. Cellular and non-cellular composites composed of a thermally curable epoxy matrix reinforced with oriented CFs were also 3D printed, showing remarkable mechanical properties and even tunable electrical and mechanical response [13,14,15,16]. By employing a direct ink writing (DIW) process that can be either UV-assisted or without UV light, wearable textile, smart materials and strain micro-structured sensors were 3D printed [17,18]. Recently, some studies reporting the additive manufacturing of fiber-reinforced thermosetting composites with continuous fibers were also carried out [19,20]. However, the high cost of CFs often limits their use in some advanced and high-performance applications, such as aerospace and automotive fields. The aerospace industry is the most important sector for carbon fiber reinforced polymers (CFRPs) with 32% of the total global demand, followed by the automotive industry, which currently represents around 21.8% of the total demand. Despite the limitations of CFs, the global demand for CFRP was estimated at 141,000 tons in 2019, with an average annual growth rate of about 10%, leading to a forecast of around 170,000 tons in 2021 [21].

To improve the low effectiveness-cost ratio of CFRPs and to reduce their environmental impact, the development of CFRPs recycling technologies is of crucial importance [22,23]. The recovery of carbon fibers from composites is a well-established practice and the pyrolysis of CFs has been classified so far as the most mature technology for the recycling of CF composites [22]. Moreover, the recovery of CFs through pyrolysis is estimated as having a much lower energy demand and carbon footprint than virgin CFs production (i.e., more than 80% decrease in the cumulative energy demand and more than 50% reduction of greenhouse gas emissions) [24]. However, recycling processes can reduce the surface and tensile properties of recycled CFs (rCFs) [25]. It is, therefore, important to explore new routes of reuse for rCFs and investigate the resulting performance of reprocessed CF composites.

Here, in this work, pyrolyzed carbon fibers underwent a sizing treatment, designed to reuse them for the realization of recycled thermoplastic composites. For a circular economy model, these already-sized CFs can offer the opportunity to reuse recycled CFRPs in different applications and extend their lifetimes. In this perspective, the pyrolyzed and sized recyclate was shredded and combined with a thermally and UV-curable resin to demonstrate the processability of rCF thermosetting composites through the UV-assisted DIW process.

The objectives of the shredding process (also called size reduction, grinding, or comminution) are mainly two: (i) to obtain high degrees of liberation of target materials from heterogeneous particles achieved by a particle made of a small number of materials and the highest possible liberation is obtained for particles made of only one material [26] and; (ii) to create homogenous particle flows (both in shape and in size) with fine controlled dimensions. This is fundamental for the direct reuse of these particles in new high added-value products. To reduce particle dimensions, it is possible to apply mechanical or non-mechanical strains (either thermal or chemical ones). Due to the low cost and reduced energy consumption, this work focuses on mechanical strains, based on pressure, shear, cut, or impact (both interparticle or with a surface) or a combination of them.

To the best of our knowledge, this is a pioneering work, which describes the use of rCFs in the 3D printing process. This approach is of great importance as it demonstrates the possibility of 3D printing self-standing structures loaded with rCFs. Although the 3D printable resin loaded with rCFs showed a low degree of UV-induced crosslinking during the DIW process, self-standing structures were successfully 3D printed thanks to the addition of a rheological modifier. A urea-modified agent was selected as a rheological modifier, taking into account previous works, where self-standing objects were 3D printed due to the formation of a hydrogen bond structure [27]. The present work shows a successful 3D printing of a curable resin loaded with recycled CFRPs. The selection of an appropriate modifier of the 3D printable resin and the use of a UV-assisted DIW process enabled the additive manufacturing of CF composites with a high elastic modulus in agreement with the theoretically expected value. A fully crosslinked material was also obtained with a post-curing cycle after 3D printing. This study paves the way for future research activities to explore the printability of freeform and complex structures as well as improve fiber-matrix adhesion and thus mechanical properties.

## 2. Materials and Methods

### 2.1. Materials

The composite matrix consisted of ethoxylate bisphenol A diacrylate resin supplied from Arkema, Colombes, France (local distributor: Came S.r.l., Lainate, Italy), and named SR349.

To initiate the polymerization by UV light, ethyl phenyl (2,4,6-trimethyl benzoyl) phosphinate, hereinafter named TPO-L, was added to the acrylic resin (Lambson Limited, Wetherby, United Kingdom). Dicumyl peroxide was also added to the matrix to increase the crosslinking degree during the thermal post-curing (Sigma-Aldrich Corporation, St. Louis, MO, USA). TPO-L and dicumyl peroxide were added to the solution with a proportion of 3 wt% and 0.3 wt%, respectively.

A solution of a urea-modified agent in amide ester (BYK-7411 ES from BYK (BYK, Wesel, Germany), and hereinafter called BYK was added to the resin system as a rheological modifier to improve the quality of the extrusion during the process. Raw components for the resin system were used as received.

Recycled CFs were obtained from a pyrolysis process performed by Tecnalia Research & Innovation Center (San Sebastian, Spain) on expired pre-impregnated (prepreg) CFs, kindly supplied by Aernnova Aerospace S.A (Minano Mayor, Spain). The expired resin of prepregs was removed by the pyrolytic treatment and, therefore, no effect on the final results, due to the use of expired prepreg CFs, was expected.

### 2.2. rCFs Sizing

For the rCF resizing, a non-ionic maleated polypropylene dispersion (Hydrosize^®^ PP2-01, Michelman Inc., Cincinnati, OH, USA) of 5 wt% solids content with deionized water was generated. The dispersion was stirred at 30 °C and for 1 h for good dispersion. The rCFs were immersed into the solution and then dried in a convection oven at 80 °C for 10–12 h and sealed in plastic bags to wait for the shredding. This procedure without any rinsing of excess sizing generates fiber agglomerates, which have a higher density than the untreated rCFs and are thus more suitable for shredding. However, the large amount of sizing on the CF surface might absorb moisture before compounding and act as a plasticizer at the fiber–matrix interface.

### 2.3. Shredding

To 3D print the thermally recycled and sized CFs, their length had to be reduced. Depending in particular on the initial dimensions of end-of-life products, it could be possible to have several size reduction steps that could be divided into coarse and fine shredding. Coarse ones reduce dimensions of products to 10–20 mm, obtaining particles that, in most cases, are not liberated. On the other hand, fine comminution can take in input coarse particles giving in output particles with a dimension from 6 mm to few tens of microns (in case of ultrafine size reduction processes). Table 1 compares the different technologies commonly used for shredding of composite materials with an indication of the throughput, underling the efficiency and highlighting the suitability for composites.

**Figure 1:**
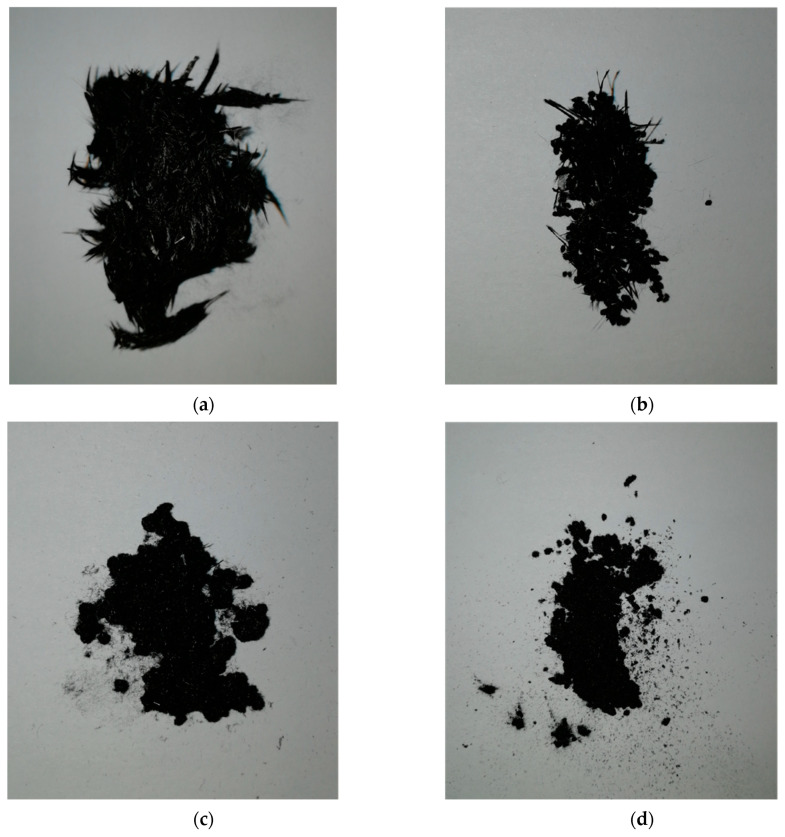
rCFs images after the shredding process and manual sieving: (**a**) rCFs sizing and shredding with the cutting mill; (**b**) rCFs fraction after the quad blade chopper shredding and sieving with a dimension higher than 300 µm; (**c**) rCF fraction with a dimension higher than 100 µm; (**d**) rCFs fraction with a nominal dimension of 100 µm.

To obtain a fine grinding of thermally recycled and sized CFs, a first shredding step with a cutting mill by Retsch GmbH (model SM-300, Haan, Germany) at a velocity of 2000 rpm with a grate size of 1 mm was performed. A fast-rotating rotor (from 700 rpm to 3000 rpm) was equipped with multiple removable inserts, usually organized in different lines. The material was loaded from the top of the chamber and was simultaneously cut by the inserts and compressed on the chamber. The material was unloaded from the bottom through a grate of variable size. Cutting, shearing, and impact effects cooperated for a homogeneous and effective size reduction of materials. This configuration is ideal for materials, which can be hard to cut to obtain a fine size (down to 0.25 mm). However, after this shredding step, it was not possible to obtain a powder material to be used for 3D printing, as shown in Figure 1a.

A further shredding step was performed to reach the dimensional requirements for the UV-DIW process [28]. To further reduce the rCF length, a quad blade chopper CH580 (Kenwood Limited, Havant, United Kingdom) was used. The first attempt was carried with 1 g of material alternating 1 min of chopping and 30 s of pause for a total of 30 min. After this first chopping, no significant difference was visible when compared to Figure 1a. To enhance the brittleness of rCFs, dry ice was inserted in the chopper, and the process was repeated with a longer cutting period, resulting in 5 min chopping and 1 min pause for a total period of 2 h. To separate the different fiber sizes obtained, a manual sieving process with 300 and 100 μm sieve mesh size was performed. Figure 1b shows the fraction with a dimension higher than 300 μm because it could not be sieved. Figure 1c and d show the two particle sizes obtained with the sieving process.

### 2.4. 3D Printing

3D printable ink formulations were obtained by mixing the resin system with a magnetic stirrer at room temperature for 2 h. Afterward, rCFs were manually incorporated into the matrix to obtain a homogeneous and printable material.

For the sake of simplicity, a nomenclature was assigned for each 3D printable ink formulation according to the “XBYAER” scheme. Specifically, X corresponds to the urea-modified agent percentage in weight, and it is followed by the first letter of its commercial name “BYK”. Y represents the filler concentration by weight of the rCFs, and it is followed by the short name “AER” of the primary supplier Aernnova. Accordingly, 6B25AER formulation contains 6 wt% of the urea-modified agent and 25 wt% of rCFs.

After the mixing, 3D printable inks were suitable for the DIW process. The starting 3D printing apparatus was based on a modified version of a commercial FFF 3D printer (3Drag supplied by Futura Group S.r.l., Gallarate, Italy). The 3D printer conversion for the DIW process was done by substituting the original filament extruder with a custom-designed syringe extruder and by modifying the firmware of the 3D printer. The custom syringe extruder and the modified firmware are available in an open-source repository [29,30]. Considering the photo-polymerization issues related to CF, other modifications were made to the system, and they will be better described in Section 3.5. The design of the custom pieces for the modified 3D printer was carried out using the CAD software Fusion 360 (Autodesk, San Rafael, CA, USA). Prusa i3 MK3S 3D printer was then used for 3D printing the models by creating a Gcode file with Slic3r PE 1.41.3 open source slicing software (Prusa Research, Prague, Czech Republic). For tensile specimens performed with DIW, Cura open source slicing software was adopted (Ultimaker B. V., Utrecht, The Netherlands).

For the present experimentation, a 20 mL syringe with a stainless-steel conic UV-shielded nozzle (1.04 mm diameter) was equipped for the system. The parameters employed during the 3D printing are listed in Table 2.

The polymerization conversion of the material should be increased using a post-curing cycle, taking into account the CF light absorption. For this reason, a UV post-curing step was carried out in a UV chamber Polymer 500 W (Helios Italquartz S.r.l., Cambiago, Italy) with a UVA emittance mercury vapor lamp type Zs (950 W/m^2^) for 15 min for each sample side. Afterward, a thermal post-curing step was performed in a non-controlled atmosphere oven at 140 °C for 2 h.

### 2.5. Material Characterization

The recyclate morphology was investigated using Scanning Electron Microscopy (SEM) micrographs performed with Cambridge Stereoscan 360 (Cambridge Instrument Company Ltd., Cambridge, United Kingdom). Secondary and backscattered electron probes were used, and physical vapor deposition of gold for 1 min was performed to prepare the sample surfaces.

**Figure 2:**
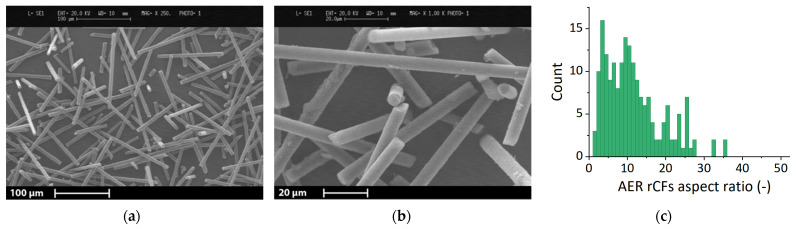
(**a**) SEM micrographs of rCFs showing the different length; (**b**) inset of the micrograph; (**c**) rCF aspect ratio distribution graph.

Furthermore, SEM micrographs were also employed for the evaluation of the carbon fiber length and diameter through MATLAB software (The MathWorks Inc., Natick, MA, USA). A specific MATLAB application was developed to automatize the measurements, and a brief description of its working principle is given below. A greyscale image is transformed into a binary image using a threshold value equal to 0.2 between 0 (black) and 1 (white). Black pixel isles inside white pixel isles and vice versa are removed using a threshold of 100 pixels as the minimum area of the inside isle. With the fibers identified by white isles, the software subdivides and rotates each isle until the length of the isle is horizontal. Further refinement of the isle is performed to obtain an almost rectangular isle. Finally, the software measures the dimension of each rectangle circumscribing the isles. This open-source application is available on a public repository [31]. The average values of the rCFs length and diameter are reported in Table 3. Figure 2 shows two representative micrographs of the rCFs and their aspect ratio distribution. Afterward, the aspect ratio distribution was used for the Halpin–Tsai model calculation for aligned fibers [32]. The prediction was performed by calculating the theoretical elastic modulus for each measured fiber substituting the aspect ratio of each fiber to the Halpin–Tsai model. Then, the values were averaged to obtain the mean Halpin–Tsai elastic modulus of the composite with the corresponding fibers aspect ratio distribution.

Rheological tests on rCFs were carried out with Discovery HR-2 (TA Instruments Inc, New Castle, DE, USA) with a 20 mm plate-plate geometry and a 1 mm gap. A 3-step oscillation strain test was performed at 1 Hz (forerun by only 3 min rest period), 0.01% strain for 20 s, 20% strain for 20 s, and 0.01% strain for 1 h. This test was specifically defined to simulate the 3D printing process and make some comparisons. In detail, the first step of the test shows the material inside the reservoir (oscillation strain quasi-zero), and the second step of the test shows the material during extrusion (the oscillation strain was fixed at 20%). Finally, the third step shows the material behavior after the deposition.

Additional analyses were performed for a better understanding of the degree of cross-linking of the 3D printable ink. As described in our previous work [33], the material was crosslinked during the 3D printing process thanks to three UV-LEDs controlled by an external unit. Consequently, the presence of a UV photo-initiator (TPO-L) is needed, and the measurements of UV crosslinking were performed by ultraviolet light differential scanning calorimetry (UV-DSC) analyses with a Mettler–Toledo DSC/823e, Mettler Toledo, USA, equipped with Lightningcure LC8, Hamamatsu Photonics, Japan. UV-DSC tests were carried out exposing the samples for 3 min to a 365 nm UV radiation (607.7 mW/cm^2^) twice. The difference in enthalpy between the first and the second runs was used to measure the UV conversion.

The glass transition temperature of the 3D printed sample after post-curing was evaluated through differential scanning calorimetry (DSC) with a Mettler-Toledo DSC/823e (Mettler Toledo, Columbus, OH, USA). The heating ramp was set from 0 °C to 250 °C with a 20°/min of heating rate.

Gel content was also evaluated to determine the quantity of cross-linked material after the post-curing treatment. After the immersion of the material in acetone for 24 h on a magnetic stirrer (with a proportion of 300 mL for 2 g), the solution was filtered, and not solubilized residual particles were collected in a paper filter (Whatman Filter Papers, Cat No 1001 125). The filtered solution was poured into a flask. The solid residue and the flask were put in a vacuum oven at 50 °C for approximately 48 h, until two consecutive weightings, after 6 h from each other, were constant. Knowing the weight of the flask and the paper filter, the final weights of the solubilized residue in the flask and the not solubilized one can be calculated, respectively. Finally, gel percentage (*Gel%*) can be estimated thanks to the equation:

(1) $$Gel\%=\;\frac{m_{sample}-m_{sol}}{m_{sample}}\times 100$$

where *m_sample_* is the starting mass of the material sample, and *m_sol_* is the mass of the solubilized residue after the procedure.

Tensile properties were evaluated with a Zwick Roell Z010 (ZwickRoell GmbH & Co. KG, Ulm, Germany) with a 10 kN cell load at a speed of 1 mm/min. For the tests, the ASTM standard test method D3039/D3039M-17 (2017) was adopted [34]. Accordingly, specimens were 3D printed with a nominal gauge length of 40 mm, a width of 10 mm, an overall length of 100 mm, and a thickness of 2 mm. Then, they were manually polished to remove the asperities and achieve a constant cross-sectional area, and their final dimension was measured.

## 3. Results and Discussion

### 3.1. Shredding

Among all the different technologies available for shredding of composite materials (Table 1), the most suitable one is the cutting mill, in particular considering continuous feed mode, enabling profitable industrial applications. Firstly, this machine allows the treatment of both thermosets and thermoplastics composites, in particular, if paired with a cyclone system (facilitating the exit of the particles from the shredding process and decreasing the internal temperature of the comminution chamber). In addition, the cutting mill, in continuous feed mode, can lead to a homogeneous distribution of particles in terms of both shape and dimension. This is possibly due to the combination of cut and compression strains and particles discharge through a grate with a specific controllable dimension.

Several factors affect process results with the cutting mill, both design and controllable parameters. The first ones are the volume of the chamber, influencing the maximum quantity of material that could be treated and the consequent throughput, the number of cutting tools, and the number of cutters per cutting tool, affecting the efficiency of the breakage process. On the other hand, the controllable parameters are the rotational speed of the rotor, impacting on the throughput and the cost of the process, the feed rate, strictly related to the throughput and the saturation of the chamber, and the grate size. This last one is the most important to reach a specific target dimension, maximizing the number of particles around a specific size.

However, after the first shredding process was performed with the cutting mill, the thermally recycled carbon fibers showed higher dimensions than the nozzle used for 3D printing. To meet the dimensional requirements for the 3D printing of reinforced composites, preliminary tests were performed with a quad blade chopper to obtain a finer recycled material. This result was already shown in Figure 1c and d. The obtained rCFs were successively measured through SEM micrographs. Micrographs of the rCFs can be seen in Section 2.5 together with the measurement results in terms of fiber length, diameter, and aspect ratio distribution (Figure 2 and Table 3). As described in the Experimental Section, the aspect ratio distribution was also used for the Halpin–Tsai model calculations.

### 3.2. Ink Formulation and Process Requirements

**Figure 3:**
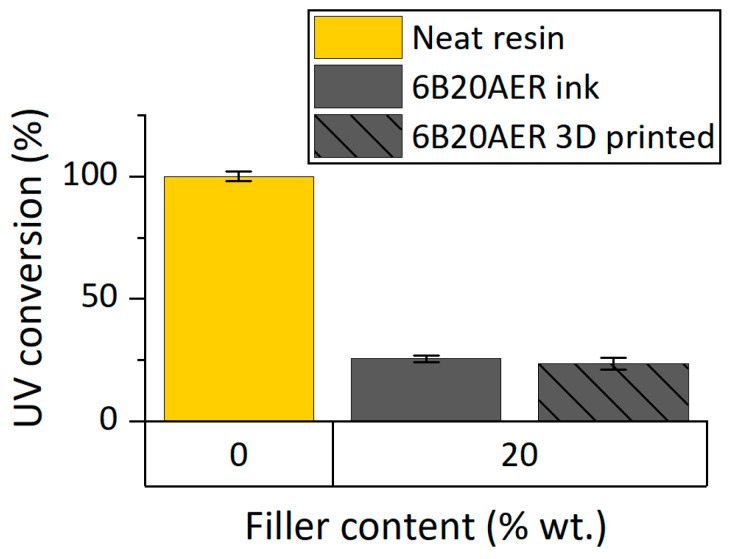
Characterization of the UV-induced crosslinking of rCF 3D printable inks: UV conversion of 3D printable inks and 3D printed specimens evaluated by UV-DSC.

Since the resin matrix is a dual-cure system, the final degree of crosslinking is the sum of the UV and thermal crosslinking. The UV source was employed to ensure the shape retention of the extruded material and stabilize it during the fabrication of the 3D printed objects layer-by-layer, whereas the thermal post-curing aims to finalize the material crosslinking. As shown in Figure 3, the UV conversion of the neat resin system and the ink containing the rCF before and after the 3D printing process was evaluated by UV-DSC.

Considering the UV conversion of the neat resin as the maximum, the ink has a very low conversion. Moreover, the conversion during the fabrication process is almost negligible since there is very little difference between the UV-DSC analysis for the ink before and after the 3D printing process. For these reasons, a change of the rCF ink formulation and a modification of the process were necessary.

### 3.3. Rheological Modification

**Figure 4:**
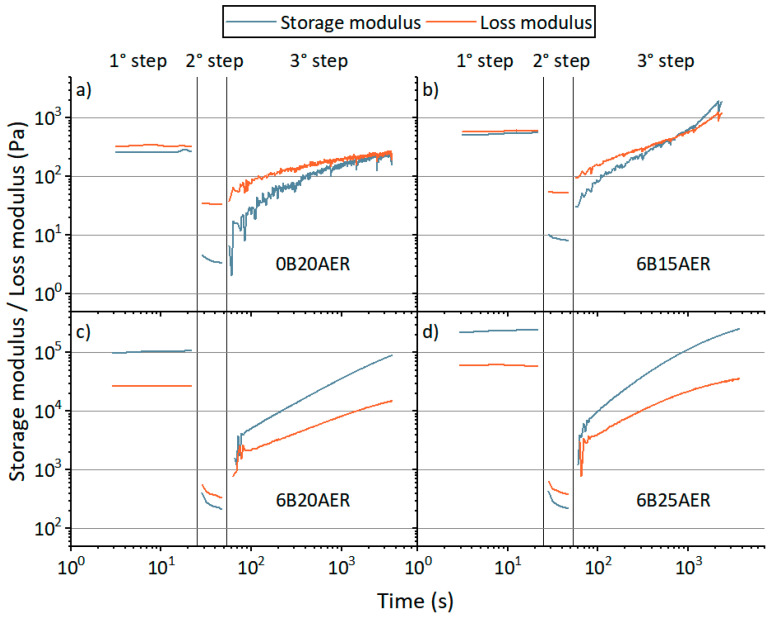
Rheological step tests for 3D printable inks composed of the ethoxylate bisphenol A diacrylate resin: (**a**) with 20 wt% of rCFs (0B20AER); (**b**) with 6 wt% of the urea-modified agent and 15 wt% of rCFs (6B15AER); (**c**) with 6 wt% of the urea-modified agent and 20 wt% of rCFs (6B20AER) and (**d**) with 6 wt% of the urea-modified agent and 25 wt% of rCFs (6B25AER).

To overcome the poor UV reactivity of the rCF-based 3D printable ink, a rheological modification was studied with the intent to achieve a correct three-dimensional deposition even with a low conversion material. This allowed shape retention to be achieved not only by the UV conversion during the 3D printing process but also by the rheological modifier into the ink formulation. The addition of a modified-urea agent was a solution already seen in the literature [27]. The influence of this rheological modifier was tested for rCF 3D printable ink, and the results are shown in Figure 4.

The addition of 6 wt% over the resin system allows for the transition between solid-like and liquid-like behavior of the ink during extrusion. Figure 4a,c show, respectively, the difference of the ink without and with the modified-urea agent, highlighting how its addition modified the ink behavior.

At rest, before and after extrusion, the ink behaves like a solid. As a consequence, the deposited material is stable and it will not lose the shape defined through the deposition process. Figure 4b–d shows the influence of different percentages of rCFs over the rheological behavior. Reducing the rCF content to 15 wt% (6B15AER), the solid-like behavior of the ink is not rapidly recovered after the extrusion, as shown in Figure 4b where G’’ is higher than G’ at the beginning of the third step of the test. The material starts to recover the solid-like behavior only after 15 min (~1000 s) of rest. Moreover, 6B15AER ink behaves like a fluid at the first step of the test. This shows that the stresses and deformations that occurred during the material loading in the rheometer plates destroyed H-bonding interactions in 6B15AER ink with the urea-based rheological modifier. The time passed before starting the experiment was not enough to reform hydrogen bonds. Increasing the rCFs content, the change from the liquid-like to solid-like behavior occurs rapidly, and the transition timeframe is comparable with the deposition process.

Considering the poor UV conversion of the material and the low difference in solid-like recovery after extrusion between 20 wt% and 25 wt% rCFs inks, 20 wt% rCFs ink was selected (6B20AER ink) for the following tests.

### 3.4. Crosslinking and Thermal Properties

The material was tested to assess the post-curing cycle performance. DSC analysis and gel content of 6B20AER formulation were evaluated according to the parameters previously described in Section 2.5. As shown in Table 4, the calorimetry analysis shows a residual enthalpy of 11.4 J/g after the post-curing cycle and a glass transition temperature of 82 °C.

The presence of a residual enthalpy could be due to unreacted crosslinking agents. Alternatively, it could be linked to the reduced mobility of the partially crosslinked material that hinders the propagation of the reaction. Nevertheless, the gel content measurement evidenced a high degree of crosslinking, confirming the good quality of the crosslinked material. Further investigations could give a more accurate explanation of the presence of possible unreacted groups.

### 3.5. 3D Printing Process

**Figure 5:**
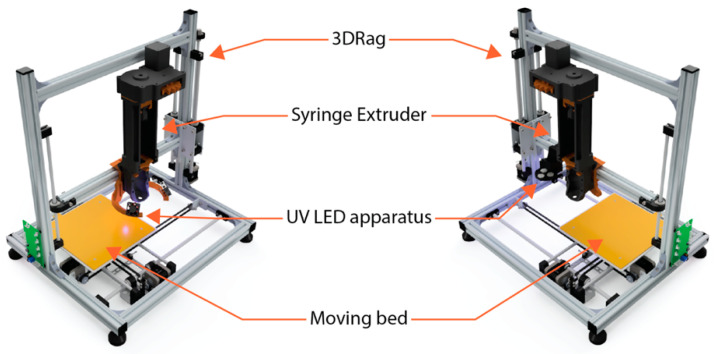
The previous version of the UV-DIW 3Drag 3D printer (on the left) and the new 3D printing layout for CF reprocessing (on the right).

As anticipated in the process requirements section, a modification of the process was investigated. The previous 3D printer setup allows the deposition of the material and, contextually, its in-situ polymerization [35]. This solution performed well with high reactivity inks, but it led to a difficult control over the 3D printed shape for inks with a low UV conversion. Figure 5 shows the previous 3D printer apparatus (on the left), and the new one (on the right). The main changes are related to the UV-LED system that was modified and positioned at the side of the extrusion head.

**Figure 6:**
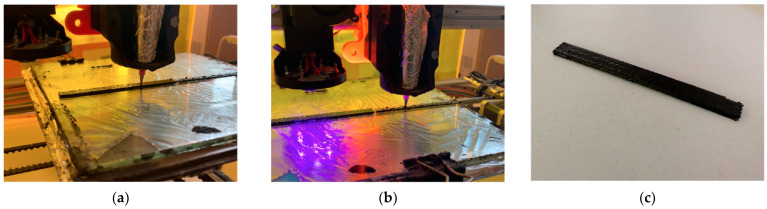
(**a**) 3D printing phase with the modified sided-LED apparatus; (**b**) UV conversion phase; (**c**) 3D printed tensile specimen with 20 wt% of rCFs.

After the deposition of each layer, the curing apparatus was moved over the deposited material and switched on. Relatively thin layers of 0.2 mm were deposited to maximize the composite crosslinking. To crosslink the material conformingly, the UV-LED apparatus was forced to move over the 3D printed object following the deposition path. This was obtained by manual modification of the Gcode file that consisted of translating the extrusion paths by a distance equal to the horizontal offset between the center of the UV-LED apparatus and the tip of the extrusion head. Figure 6a,b show the deposition step and the curing step, respectively. The generation of the Gcode file can be optimized for complex objects which require longer Gcode files. However, for the generation of the Gcode in this work, a manual modification of the file was performed because it was easier due to the simple deposition path needed for the fabrication of a tensile specimen. The result of the 3D printing process is shown in Figure 6c. A total of five tensile test specimens were successfully fabricated.

### 3.6. Mechanical Properties

**Figure 7:**
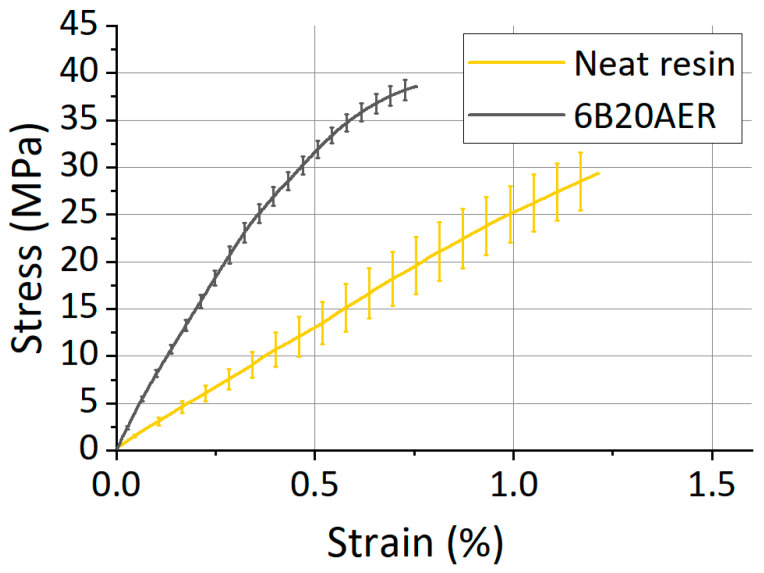
Mechanical behavior of 20 wt% CF formulation (6B20AER) and neat resin: Stress versus Strain graph (average curves).

Afterward, the tensile properties were tested according to the main parameters described in Section 2.5. The averaged stress vs strain curve measured for the rCFs remanufactured material is shown in Figure 7 together with the averaged curve of the neat resin.

The relatively low viscosity and the Newtonian behavior of the neat resin were not suitable for the 3D printing process. Consequently, the specimens of the neat resin were produced by casting and their tensile properties were shown in a previous work [33]. The measured elastic modulus of the neat resin was employed for the calculation of the theoretical Halpin–Tsai elastic modulus of the composites and the strength of the resin was considered as a reference for matrix properties of the composite material in this study.

Moreover, although the neat resin specimens were obtained with a different technology for the above-mentioned reasons, some general considerations can be made. A brittle behavior was observed for both the neat resin and the remanufactured composite. As visible in Figure 7, a quite high increase in stiffness for the rCF composite sample was achieved in comparison with the neat resin. Despite the elongation at break is lower than the neat resin, the tensile strength value increased and the toughness of the remanufactured composite material is higher than the matrix. The mechanical properties of the two materials are reported in Table 5.

The elastic modulus of 3D printed recycled composites was three times higher than that of the neat resin. The theoretical elastic modulus, calculated with the Halpin–Tsai model for aligned fibers and using the average aspect ratio of the rCFs, was 8.6 GPa. Considering the high distribution of the rCFs aspect ratio (Table 2), the theoretical value of the elastic modulus presented here (8.1 ± 2.2 GPa) is an average value with a standard deviation obtained from the aspect ratio of each fiber. This theoretical value and the experimental value of elastic modulus are in very good agreement (Table 5).

Compared to other 3D printed thermosetting composites reinforced with virgin CFs [14,16], the 3D printed composites, developed in this work and obtained from rCFs, exhibited a comparable average value of elastic modulus. For this reason, these 3D printed rCF composites can be considered as a good alternative to virgin CFRP for several fields of application (i.e., automotive). Tensile strength of 3D printed composites reinforced with rCFs was slightly lower than values measured for other additive manufactured composites composed of epoxy matrix loaded with virgin CFs. However, this can be due to the epoxy matrix, which presented a higher tensile strength (80–85 MPa) than the acrylic matrix used in this work (26 MPa) [16].

### 3.7. Scanning Electron Microscopy (SEM) Analysis

**Figure 8:**
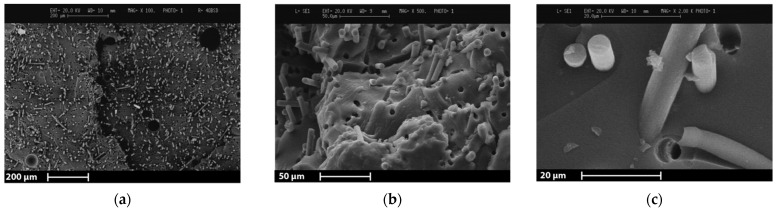
SEM micrographs of 3D printed tensile specimen cross-sections with 20 wt% CF formulation (6B20AER): (**a**) details on voids and overall fibers distribution in the matrix; (**b**) fibers detachment from the matrix; (**c**) fiber interactions with the matrix.

Fracture surface analysis confirmed the preferential alignment of the fibers, which is longitudinal to the 3D printing direction and perpendicular to the fracture surface. The 3D printing direction was in fact longitudinal to the specimen. The preferential fiber alignment was also observed in other studies, which reported the reinforcement of a 3D printed thermosetting epoxy matrix with short CFs [36]. 3D printing process parameters such as fiber content and fiber length were found to significantly affect the degree of fiber alignment and the effect of these parameters will be investigated in future works regarding the 3D printing of rCFs. As shown in Figure 8a, the matrix homogeneity evidences the absence of 3D printing marks between the deposited layers. However, some voids related to entrapped air bubbles were detected. Figure 8b,c shows the exposed fibers with a clean surface and the holes left by the pulled-out fibers. Accordingly, fibers pullout can be stated as the predominant failure mode of the material.

## 4. Conclusions

This paper demonstrates the feasibility of the 3D printing of rCF thermosetting composites. Thermally recovered CFs can be reprocessed to obtain a reinforcing filler for photo- and thermally curable 3D printable inks. Moreover, this work reveals a high potential for the use of thermally recycled carbon fibers shredded with a frugal approach, even if a systematical design of the shredding process is needed. This will be investigated in future works through an extensive experimental campaign with innovative shredding machines as an ultra-centrifugal comminution system. Thanks to the addition of a urea-modifier agent, the poor UV reactivity of the ink due to the rCF presence is not an obstacle for the UV-assisted 3D printing process. Shape retention was achieved thanks to a new 3D printing apparatus that separates the UV crosslinking phase from the material extrusion for each 3D printed layer. Future works on applications, case study objects, and complex models could further prove the potentiality of decoupling the material deposition by the material crosslinking processes. Considering the mechanical properties, the addition of rCFs into the matrix resulted in a noticeable increase in toughness and elastic modulus. The latter values confirmed the theoretical Halpin–Tsai prediction, showing an overall quality of the UV-assisted 3D printing process. Nevertheless, the toughness of the material could be furtherly increased by specifically designing a sizing agent. In this way, the interfacial adhesion of the sized rCFs with the resin used in this work could be higher, leading to better mechanical performance. Although the sizing agent was designed for rCFs composites with a polypropylene matrix, the exploitation of these rCFs was investigated in a circular economy perspective and good mechanical properties were obtained in this paper. From these promising results, 3D printed rCF reinforced thermosetting materials can potentially lead to new technology applications in circular economy models, substantially reducing the economic and environmental impacts of the composite manufacturing sector on a global scale.

## Figures and Tables

**Table 1:** Comparison of the different shredding technologies commonly used for composites comminution.

Technology	Type of Shredding	Throughput	Efficiency	Suitability for Composites
Two shafts shear shredder	Coarse	High	Risk of fibers wrapping	Good for coarse shredding
Single shaft shear shredder	Coarse	High	Good	Good for coarse shredding
Hammermill	Coarse/fine	High	Good for thermoset matrixes, not suitable for thermoplastic matrixes	Good for coarse shredding of thermoset composites
Impact crusher	Coarse	High	Not suitable for thermoplastic matrixes	Low
Jaw crusher	Coarse	High	Not suitable for thermoplastic matrixes	Low
Cutting mill	Fine	Mid	Good	Good for fine shredding
Disc mill	Fine	Low	Not suitable for thermoplastic matrixes	Low
Chain mill	Coarse	High	Good for thermoset matrixes, not suitable for thermoplastic matrixes, creation of powder	Good for coarse shredding of thermoset composites

**Table 2:** 3D printing parameters for rCF composites with 20 wt% of rCF and sided LEDs position.

Parameters	6B20AER
Perimeter number (-)	0
Infill (%)	100
Raster angle (°)	0
Flow (%)	100
Speed (mm/s)	12–15
Layer Height (mm)	0.20
Nozzle Diameter (mm)	1
LED UV (-)	3 × 3 W (395 nm-lateral)

**Table 3:** Average values of carbon fiber length, fiber diameter, and aspect ratio for rCF.

Filler	Length (µm)	Diameter (µm)	Aspect Ratio (-)
rCF	74.6 ± 48.3	6.5	11.5 ± 7.4

**Table 4:** Residual enthalpy, glass transition temperature (T_g_), evaluated by DSC tests, and gel percentage (*Gel%)*, measured by gel content tests, for rCF samples with 6 wt% of rheological modifier and 20 wt% (6B20AER).

3D Printable Ink	Residual Enthalpy (J/g)	T_g_ (°C)	*Gel%* (%)
6B20AER	11.4	82	99

**Table 5:** Mechanical properties of 20 wt% CF formulation (6B20AER) and neat resin: Elastic Modulus and Halpin–Tsai model, Tensile Strength, Elongation at break, and Toughness.

Formulation	Layer Height (mm)	Elastic Modulus (GPa)	Halpin–Tsai Prediction (GPa)	Tensile Strength (MPa)	Elongation at Break (%)	Toughness (J/mm^3^)
Neat Resin	n/a	2.6 ± 0.4	n/a	25.9 ± 1.2	1.0 ± 0.2	136.6 ± 25.0
6B20AER	0.20	8.1 ± 0.5	8.1 ± 2.2	39.4 ± 2.1	0.9 ± 0.1	218.6 ± 57.2

## Data Availability

Publicly available datasets were analyzed in this study. The data can be found here: [https://github.com/piuLAB-official/Dataset_A.Mantelli_2021_Polymers] (accessed on 27 February 2021). If you will use the data, please cite them in the following way: [dataset] Andrea Mantelli, Alessia Romani, Raffaella Suriano, Marco Diani, Marcello Colledani, Essi Sarlin, Stefano Turri and Marinella Levi. 2021. UV-assisted 3D printing of polymer composites from thermally and mechanically recycled carbon fibers; https://github.com/piuLAB-official/Dataset_A.Mantelli_2021_Polymers (accessed on 27 February 2021).
